# Galactosaminoglycans: Medical Applications and Drawbacks

**DOI:** 10.3390/molecules24152803

**Published:** 2019-08-01

**Authors:** Vitor H. Pomin, William P. Vignovich, Alysia V. Gonzales, Ariana A. Vasconcelos, Barbara Mulloy

**Affiliations:** 1 Department of Biomolecular Sciences, School of Pharmacy, University of Mississippi, Oxford, MS 38677-1848, USA; 2 Research Institute of Pharmaceutical Sciences, School of Pharmacy, University of Mississippi, Oxford, MS 38677-1848, USA; 3 Institute of Medical Biochemistry Leopoldo de Meis, Federal University of Rio de Janeiro, Rio de Janeiro, RJ 21941-590, Brazil; 4 Imperial College, Department of Medicine, Burlington Danes Building, Du Cane Road, London W12 0NN, UK

**Keywords:** Carbohydrate-based drug discovery, chondroitin sulfate, dermatan sulfate, fucosylated chondroitin sulfate, galactosaminoglycans

## Abstract

Galactosaminoglycans (GalAGs) are sulfated glycans composed of alternating *N*-acetylgalactosamine and uronic acid units. Uronic acid epimerization, sulfation patterns and fucosylation are modifications observed on these molecules. GalAGs have been extensively studied and exploited because of their multiple biomedical functions. Chondroitin sulfates (CSs), the main representative family of GalAGs, have been used in alternative therapy of joint pain/inflammation and osteoarthritis. The relatively novel fucosylated chondroitin sulfate (FCS), commonly found in sea cucumbers, has been screened in multiple systems in addition to its widely studied anticoagulant action. Biomedical properties of GalAGs are directly dependent on the sugar composition, presence or lack of fucose branches, as well as sulfation patterns. Although research interest in GalAGs has increased considerably over the three last decades, perhaps motivated by the parallel progress of glycomics, serious questions concerning the effectiveness and potential side effects of GalAGs have recently been raised. Doubts have centered particularly on the beneficial functions of CS-based therapeutic supplements and the potential harmful effects of FCS as similarly observed for oversulfated chondroitin sulfate, as a contaminant of heparin. Unexpected components were also detected in CS-based pharmaceutical preparations. This review therefore aims to offer a discussion on (1) the current and potential therapeutic applications of GalAGs, including those of unique features extracted from marine sources, and (2) the potential drawbacks of this class of molecules when applied to medicine.

## 1. Introduction

**Figure 1:**
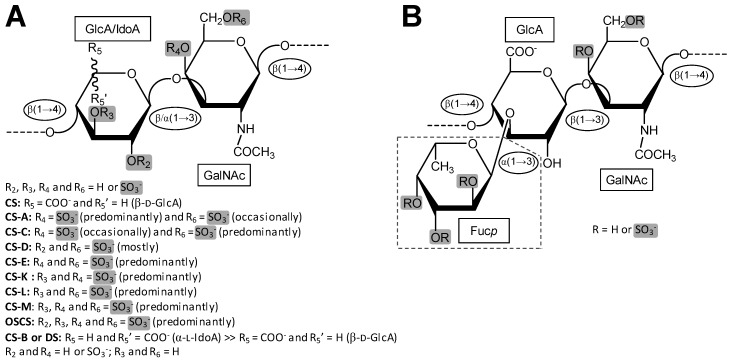
(**A**) Representative repeating disaccharide unit of chondroitin sulfate (CS) structures. CSs are composed of alternating 4-linked β-d-glucuronic acid (GlcA) and 3-linked β-d-*N*-acetylgalactosamine (GalNAc) units. While CS-A is mostly 4-sulfated at the GalNAc units, CS-C is predominantly 6-sulfated [7]. While CS-D is composed of 2-sulfated GlcA and 6-sulfated GalNAc, CS-E is composed of 4,6-di-sulfated GalNAc. CS-K, CS-L and CS-M are all sulfated at C3 position of GlcA, and respectively sulfated at positions C4, C6, and simultaneously at C4 and C6. Oversulfated chondroitin sulfate (OSCS) is fully sulfated. Dermatan sulfate (DS) also known as CS-B is composed of alternating 4-linked α-L-iduronic acid (IdoA) and 3-linked β-d-GalNAc units. (**B**) Representative repeating trisaccharide unit of the fucosylated chondroitin sulfate (FucCS). Besides consisting of the regular CS disaccharide unit as shown at panel A, the FucCS also exhibits branching units of α-l-fucopyranose (Fuc*p*) 3-linked to the GlcA unit as highlighted with the box in dashed lines. The detailed structure of the Fuc*p* unit varies according to the holothurian species. In all panels the glycosidic bonds are indicated in ellipses whereas monosaccharide types are indicated in continuous black rectangles. For fast notation of the sulfation patterns, sulfation sites are highlighted by light-grey rectangles.

Galactosaminoglycan (GalAG) is a term specifically used to designate the classes and sub-classes of glycosaminoglycans (GAGs) that contain *N*-acetylgalactosamine (GalNAc) units as the hexosamine type in their repeating disaccharide building blocks. In addition to the GalNAc unit, the disaccharide blocks of these high molecular weight (MW) polymers are additionally composed of an uronic acid, which can be either β-d-glucuronic acid (GlcA) or its C5 epimer, α-l-iduronic acid (IdoA) [1]. Although the GalAGs are homogeneously composed of alternating 3-linked GalNAc and 4-linked IdoA/GlcA units, the backbones of these glycans are extensively modified by different levels of C5 epimerization [2], sulfation contents and patterns [3,4], and in certain cases, fucosylation [5,6] (Figure 1). As detailed below, the fucose decoration on the GalAG backbone can be further sulfated at different positions and degrees [6]. The principal representatives of GalAGs are the chondroitin sulfates (CSs), dermatan sulfate (DS) and fucosylated chondroitin sulfate (FucCS) (Figure 1). Like all GAGs, GalAGs are key structural players of living organisms and are found more commonly in the extracellular matrix (ECM) environment or attached to the cell surface than inside the cells.

The structural diversity of GalAGs directly reflects their great number of biological roles and medical uses. Examples of this wide spectrum of biological roles are cell growth and differentiation [8,9], morphogenesis [10,11], cell migration [12,13] and microbial infection [14]. Examples of medical effects in which these molecules participate are anticoagulation [15,16,17,18,19], antithrombosis [20,21], anti-inflammation [22,23,24], anticancer [24,25,26], antiviral [27,28,29,30], anti-angiogenesis [31,32] and antimalaria [33], in addition to some beneficial effects in events of hemodialysis [34], cellular growth modulation [35], fibrosis [36], hyperglycemia [37] and diabetes-related nephropathy [38]. Due to the many biological and medical functions of GalAGs, several scientific groups in the world are now conducting research on these molecules. Consequently, the number of publications regarding GalAGs has increased considerably in the last three decades. This growing interest in research about GalAGs has been accompanied by the boom of glycomics [39]. The main research goals of the GalAG-related works have been the isolation and structural characterization of novel compounds, screening of new biological and medical functions, advanced structural analysis, development of novel and more efficient sequencing and analytical methods, and glycoengineering processes for partial or full chemical synthesis of GalAG oligosaccharides in the laboratory. The achievements made in these works have enabled great progress concerning the details underlying the biomedical functions and physicochemical properties of GalAGs and placed these glycans in a special position in medicinal glycomics.

However, as opposed to the many articles that have been published so far reporting the beneficial effects of GalAGs in healthcare, a few, but key, studies are now exposing the lack or potential negative effects of these molecules. In this review, we not only offer an up-to-date overview regarding the main chemical, biological and clinical aspects of GalAGs from both common and marine sources, but also highlight the downsides of these glycans regarding their current and potential medical uses. This subject is worthy of special attention from the scientific community. Hence, it is worth discussing here the facts associated with the issues related to possible impurities during manufacturing, actual effectiveness and safety during the application of GalAGs as pharmaceutical ingredients, and their potential harmful effects already pointed out in some basic studies.

## 2. CS

### 2.1. Overview

**Figure 2:**
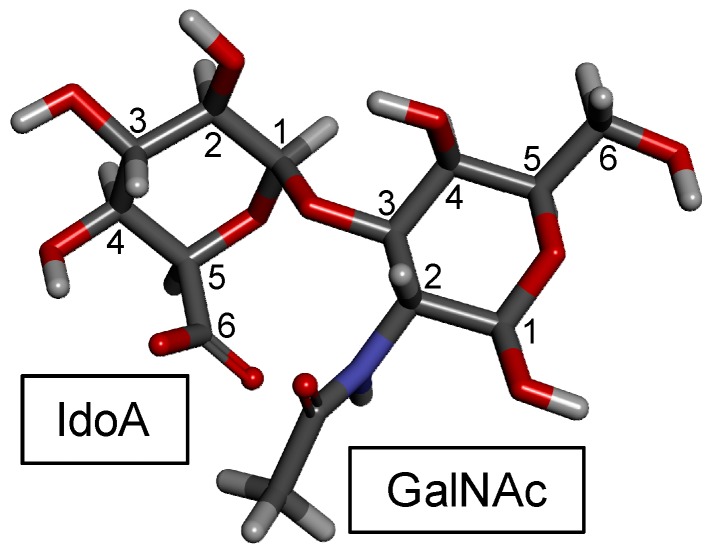
Representative disaccharide unit, GlcA(β1−3)GalNAc, of CS backbones in the 3D stick model. Hydrogens, carbons, oxygens and nitrogens are respectively represented in light grey, dark grey, red and blue. Carbon atoms are numbered according to their positions in the ring. Monosaccharides are indicated accordingly. Model created by the online AMBER/GLYCAM-based carbohydrate builder [40].

CSs are a group of GalAGs composed of alternating β-d-GlcA and β-d-GalNAc units (Figure 1A) polymerized within relatively large (50–100 kDa) and heterogeneous chains consisting of a non-uniform distribution of different disaccharide units [3]. Figure 2 illustrates the 3D structure of this disaccharide building block of the CS backbone without characteristic sulfation. The disaccharide units of CSs are further sulfated and the consequent classification of the different CS types relies primarily on the variation of the different resulting sulfation patterns. Sulfation occurs on the hydroxyl groups on the C3 and C2 positions of the β-d-GlcA and C4 and C6 of the β-d-GalNAc (Figure 1A). The type of the CS polymer is defined by the major composing disaccharide building block although hybrid compositions are more common.

The different CS polymers are found in a species and tissue-specific manner and are correlated with their biological functions once sulfation patterns are known to impact directly on activity [41]. CSs are ubiquitously expressed in vertebrates but can also be found in invertebrates [42,43,44,45], especially marine species, which express CSs with unusual sulfation patterns [46,47] (Table 1). A case of a CS with a rare sulfation pattern not included in Table 1 is the CS found in the head of the shrimp *Litopenaeus vannamei*. This CS is disulfated at C2 and C3 positions of the GlcA unit [48]. As a main component of the ECM, CSs possess a diverse set of physiological functions, especially those necessary for tissue development and repair [49,50], inhibitory and stimulant controlling effects over the central nervous system [51,52,53], and maturation of the organisms [8,9]. Due to these general properties, CS has been widely exploited as an active ingredient in over-the-counter healthcare supplements, mainly for easing issues associated with osteoarthritis [54], especially joint (knee) pain and inflammation [55,56,57].

A common and rich biological source of CS is the cartilaginous tissues. CS-based dietary supplements are basically derived from terrestrial sources, which are easy to obtain as a byproduct of commercial agriculture from both bovine trachea and porcine (ear and nose) tissues, but shark fin, ray, crocodile and chicken keel are not entirely uncommon [63]. This, however, is a limiting approach to the utilization of these compounds as therapeutic drugs because of the lack of diversity within structures of the terrestrial sources which often contain mostly CS-A with lesser amounts of CS-C subunits [63]. In addition to the lack of chemical diversity, there is also a concern with safety associated with some oversulfated derivatives of these more traditional sources after the “*Heparin crisis*” of 2018 [64]. In fact, as discussed in more detail below, impurities, although harmless, have been reported in CS-based pharmaceutical preparations destined for oral administration [65]. All these factors have contributed to encouraging research for other CS sources of more diverse structural expression as well as safer manufacturing processes. Marine animals are exceptionally promising sources of more novel CS structures with unique sulfation patterns that are either not present or present in irrelevant amounts in terrestrial organisms. In addition to that, the marine sources have provided a robust safety profile, economic utilization and more ecological use of bycatch [46,47].

**Figure 3:**
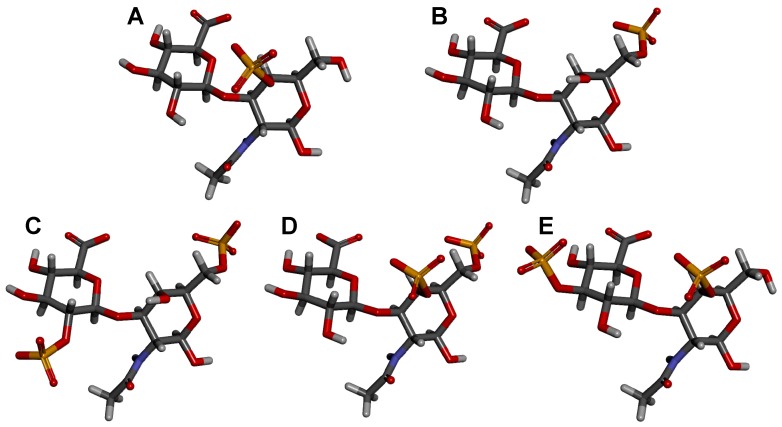
Representative disaccharide units of CS types, (**A**) CS-A, (**B**) CS-C, (**C**) CS-D, (**D**) CS-E, and (**E**) CS-K in their 3D stick models. Table 1 displays the structural description of these disaccharides. Hydrogens, carbons, oxygens and nitrogens are respectively represented in light grey, dark grey, red and blue. The carbon numbering and monosaccharide units are the same as shown in Figure 2, since all structures are the GlcA(β1−3)GalNAc disaccharide backbone. Models created by the online AMBER/GLYCAM-based carbohydrate builder [40].

Although CS-A and CS-C (Figure 3) are still expressed in marine organisms, they do not possess the same observed and potential therapeutic applications outside of more commonplace use in osteoarthritis as the other CS sub-types (Figure 1A). CS-D, CS-E, and CS-K (Figure 3), and very rarely CS-L and CS-M (Table 1) have been shown to exhibit an array of other biomedical activities related to anti-inflammation [66,67], cancer and metastasis [25,68,69], neurology [52], and antipathogenesis [28]. These CS types are less commonly found than CS-A and CS-C in mammals, and more common in marine organisms (Table 1), but indications exist of their presence, although in minor portions, in tissues of terrestrial mammals [54,70,71].

The functions promoted by CS, regardless of the CS type, are ultimately related to the activity of functional GAG binding proteins (GBPs) [72]. The biological functions of these proteins are modulated by interactions with GAGs (GalAGs), which must contain the necessary structural moieties for binding and consequential regulation [73,74], mainly sulfation patterns. The optimal systemic concentration of the active carbohydrate is another crucial requirement [75,76].

### 2.2. Biomedical Properties

#### 2.2.1. Inflammation

CS’s effects on inflammation have mostly been described in its usages in patients with osteoarthritis [22,23,77]. However, anti-inflammatory actions in multiple organs or systems, including the skin, liver, and digestive systems, have also been reported [78,79]. These effects are highly mediated by CS’s interactions with pro-inflammatory enzymes and transcription factors related to attenuation of inflammatory response [78]. Jeong-Sook Noh et al. demonstrated that in hepatic tissues CS-E produced noticeable protection against inflammatory and oxidative damage by lipopolysaccharides through down-regulation in TNF-α, IL-1β, COX-2, and iNOS hepatic inflammatory factors [66].

H. Kawashima et al. determined that CS-E has a strong binding affinity to both L- and P- selectins, as well as some chemokines [67]. The resultant CS interactions effectively produce inhibition of the activity of these proteins in recruitment of leukocytes to the inflamed sites during events of infection and inflammation. While this is an important immune response in infection prevention and in certain controlled inflammatory processes, it is also a key contributor in the pathology and progress of multiple autoimmune diseases like psoriasis and bowel disease [80]. The balanced activity of the inflammatory events is required to restore systemic homeostasis and this stage is very commonly not achieved after some auto-immune responses or pathological inflammatory processes. In these uncontrolled events of immune and/or inflammatory responses, medical intervention is needed to down-regulate the processes. CSs of various sulfation patterns have been a key agent in these complications [22,23,66,77,78,79].

Identification of the mechanisms by which the active carbohydrate-protein complexes are regulated and stabilized, and consequently how they function during the different stages of disease, is crucial to understanding the underlying biochemistry and pharmacology of GalAGs, and to fully exploring their medical properties, especially the CSs with diverse sulfation patterns (Figure 3, Table 1). In this regard, Xu Wang et al. characterized by advanced nuclear magnetic resonance (NMR) methods the binding properties of two CS hexasaccharides: one entirely 4-sulfated at the three composing disaccharides (CS444), and one with 6-sulfation only at the non-reducing end disaccharide (CS644), in complex with the pro-inflammatory CCL5/RANTES chemokine [81]. This slight change of sulfation patterns was enough to cause big changes in the interactions. The CS444 was shown to be the best ligand for interaction and consequential structural mapping of the CCL5:CS hexasaccharide complex.

#### 2.2.2. Cancer and Metastasis

CS has, however, been shown to interact with pathways that enhance cancer growth and metastasis. Native CS-A has been shown to participate in proliferation and spread of some cancers [25]. CS-E, and likely other highly sulfated derivatives of CS, can, for example, negatively regulate the pro-tumorigenic Wnt/beta-catenin-Collagen 1 axis in breast cancer cells [82]. Inhibition of L- and P-selectins may have potential applications in treatments of some cancers where carcinoma cells bind platelets through recognition of the selectins and chemokines. Binding of platelets is an aspect of cancer metastasis that sequesters cancer cells from the blood to tissues [83,84]. Inhibition of selectins and chemokines can reduce lethality associated with certain cancers as these factors directly contribute to both the proliferation of tumor cells (cancer growth) and spread (metastasis). The use of exogenous CSs shows the ability to compete with native CSs that are involved in the development of cancer in different stages of progress of this devastating disease [25,68,69,82].

#### 2.2.3. Neural Growth Stimulation and Inhibition

CS plays a key role in regulating processes during the development of the central nervous system [51]. CS’s activity is spatiotemporally dependent, acting as either stimulator in certain cases [85,86] or as inhibitor in others [86,87]. CS, together with keratan sulfate (KS), is necessary in the high-MW molecular assembly aggrecan to inhibit neurite outgrowth [88,89]. Curiously, of particular biological importance is the potential exogenous use of highly sulfated CS-E and CS-D to bind and inhibit neural growth factors that elicit outgrowth of hippocampal neurites [52]. The more sulfated CSs, like CS-D and CS-E, show mechanisms that are likely mediated through interactions between CS and midkine, a GBP that promotes growth, survival, and other activities in target cells [90]. Ueoka et al. showed that midkine binding of CS-E is comparable to heparin, as well as to CS-K [90]. The reasons for the similar binding properties of the different highly sulfated GAGs was further clarified. In the work of Maeda et al. [91], it was shown that the binding of CS to pleiotrophin, a GBP highly homologous to midkine, is regulated by highly sulfated sequences of approximately 20 monosaccharides in length. Pleiotrophin binds with good affinity to 6B4-PG/phosphacan, a highly expressed CS proteoglycan that is present in the brain [92,93]. Exogenous CS-E can compete with the 6B4-PG/phosphacan-CS binding [94]. This highlights the role of more sulfated CS units in the binding process and regulatory events. In certain cases, the inhibitory activity of CS in neural repair can be also attenuated with the use of digestive enzymes capable to cleave CS, such as chondroitinase [95]. Careful design of this proposed enzymatic therapy will be necessary to avoid potential disadvantages, as non-selective CS lyase activity will be unable to distinguish between CS chains which play an inhibitory role in the neural repair and those involved in homeostatic functions of the neural system.

#### 2.2.4. Antiviral and Antibacterial Activity

The antimicrobial activity of GalAGs has been explored extensively, and for CS, results are promising regarding both antiviral and antibacterial applications. CS-E has displayed significant antiviral activities in all serotypes of Dengue fever [27]. CS-E has reduced infectivity of this virus through a similar action to heparin via interactions with the virus’ envelope proteins. It was observed that CS-D does not lead to the same quality of interactions, showing that in this case the position of sulfation is more important than the overall degree of sulfation [27]. It has also been shown that squid CS-E can produce antiviral activity for herpes simplex virus 1 and 2 with much higher success than heparin. The mechanism of action that is proposed is through a competition between CS-E and the attachment of the virus on the host cell’s GAG receptors [28]. Due to the relatively high MW of squid CS-E, this GalAG was not able to reduce the cell-to-cell spread within the monolayer of densely growing cells [28]. CS was also proposed as a new therapeutic target for modulating infection by *Borrelia burgdorferi*, the bacterium causative of lyme disease. This pathogen has a GAG binding site that is crucial for its spread through the tissues and consequential states of the disease related to the development of lyme arthritis [96].

## 3. DS

### 3.1. Overview

**Figure 4:**
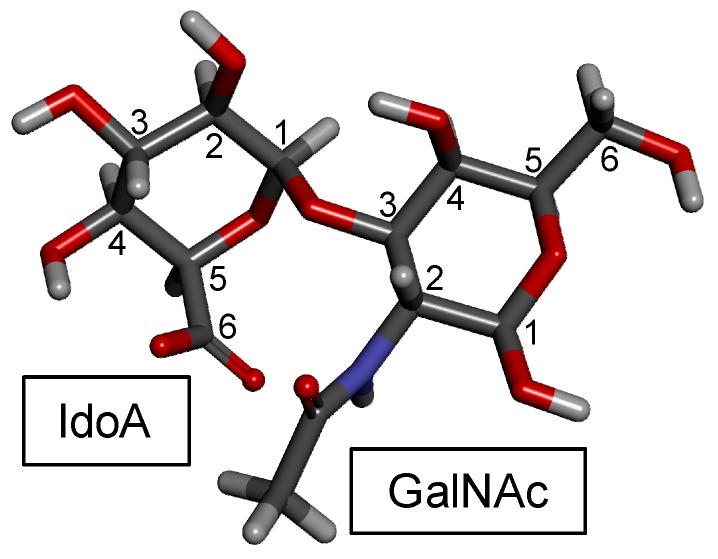
Representative disaccharide unit, IdoA(α1−3)GalNAc, of DS backbones in the 3D stick model. Hydrogens, carbons, oxygens and nitrogens are respectively represented in light grey, dark grey, red and blue. Carbon atoms are numbered according to their positions in the ring. Monosaccharides are indicated accordingly. Model created by the online AMBER/GLYCAM-based carbohydrate builder [40].

DS, also denominated CS-B, was first isolated in 1941 from porcine skin [97,98]. DS is found relatively easily but is not as common as CS-A and CS-C in animals. It is an important component of the ECM of the connective tissues. Like the CS backbone, DS is structurally composed of disaccharide building blocks, in this case of 4-linked IdoA and 3-linked GalNAc units, although GlcA units may also occur. The presence of the C5 epimerized IdoA in DS differentiates it from CS [99] (Figure 1). The 3D model of this building block is shown in Figure 4.

As for CS, the composing monosaccharides of DSs are differentially sulfated to varying degrees and positions depending on the tissues [100], cells [99] and pathophysiological conditions from which they are extracted [101]. Table 2 shows a few examples of DS sub-classes. For example, GalNAc units are mostly sulfated at the C4 position (~95%), but small amounts of sulfation (~15%) can also occur at different positions such as the C6 position of GalNAc and (~10%) at the C2 position of the IdoA [102,103]. The marine sources of DS, like for CSs, also offer unique structures to this GalAG. Interesting representatives are the DSs isolated from different species of ascidians (Urochordata) presenting distinct patterns and proportions of sulfation [99,104,105,106].

Like the diverse set of CSs with different sulfation patterns (Table 1), the structural heterogeneity of the DS molecules (Table 2) also promotes diversified biological functions [72,99], and contributes to the variety of biomedical roles played by DS. The ability of this GalAG to bind to and regulate multiple ECM proteins that are functional in numerous pathophysiological systems is also directly impacted by sulfation content and pattern [107]. Below we discuss some of the major biomedical roles played by DS.

### 3.2. Biomedical Properties

#### 3.2.1. Coagulation and Thrombosis

Among the various biological properties attributed to DS, the main one is the ability to bind to and regulate heparin cofactor II (HCII), therefore catalyzing its inhibitory effect on thrombin (IIa) [15,113]. This activity promoted by DS has been intensely investigated in terms of its use as a new therapeutic agent in coagulation and thrombosis [106,114,115]. Studies in the early 2000’s reported on the potential anticoagulant/antithrombotic use of intimatan (dermatan 4,6-*O*-di-sulfated) [116,117,118,119,120,121]. The influence of disulfated disaccharide clusters on the HCII-mediated IIa inhibition by DS of different origins has been also evaluated [122]. Quantitation of disulfated disaccharide units, including active binding sites for in HCII interaction, in DS has been performed via NMR [123] Research has also been carried out on the potential anticoagulant properties of DS-derived oligosaccharides of well-defined chemical structures [124,125], and low-MW derivatives [126], as well as on molecules with unique structures derived from marine organisms [18,104].

**Figure 5:**
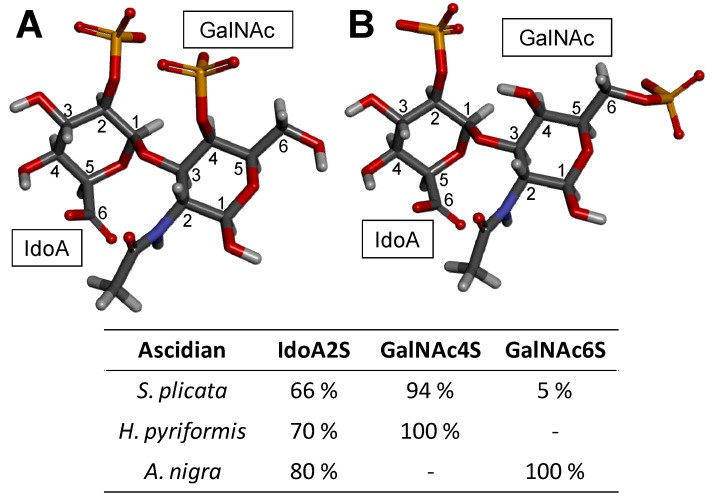
Representative disaccharide units, (**A**) IdoA2S(α1−3)GalNAc4S and (**B**) IdoA2S(α1−3)GalNAc6S, of the DS backbones from three ascidian species in their 3D stick models. These ascidian DS are highly sulfated at the C2 position of the α-L-IdoA units but differ in the pattern of sulfation at the β-d-GalNAc units. (**A**) In the species *S. plicata* and *H. pyriformis* the GalNAc moieties are heavily 4-*O*-sulfated, (**B**) whereas in *A. nigra*, they are 6-*O*-sulfated. Percentage of sulfation/site/species is shown in the table. Hydrogens, carbons, oxygens and nitrogens are respectively represented in light grey, dark grey, red and blue. Carbon atoms are numbered according to their positions in the ring. Monosaccharides are indicated accordingly. Model created by the online AMBER/GLYCAM-based carbohydrate builder [40].

About this latter case for instance, while the DS isolated from *Ascidia nigra* is fully sulfated at the C6 position of the GalNAc unit (100%) and mostly at the C2 position of the IdoA (80%) (Figure 5), the *Styela plicata* and *Halocynthia pyriformis* DSs are less sulfated at the C2 position of IdoA (65 and 70%, respectively) and widely sulfated at the C4 position of GalNAc (95 and 100%, respectively) [102,104] (Figure 5). This distinct pattern of sulfation directly impacts on the anticoagulant outcome of the ascidian DS [18,104]. While the DS from *A. nigra* has no significant anticoagulant activity, the DSs from *S. plicata* and *H pyriformis* show 4 and 5–6 times greater anticoagulant activities than mammalian DS comparatively in terms of their specific activities (units/mg) obtained by the activated partial thromboplastin time assay [104]. Regarding their potencies to catalyze the HCII inhibition of IIa, the DS from *A. nigra* shows negligible activity, while the DSs from *S. plicata* and *H pyriformis* have ∼10-fold and ∼6-fold higher activity than the native and oversulfated mammalian DS, respectively [104]. This is a clear example of sulfation pattern of GalAGs impacting biological activity. The difference in the sulfation sites of GalNAc units, 4-*O*- versus 6-*O*-sulfation, although very close in terms of content, is enough to lead to a complete different anticoagulant outcome.

Because DS is an effective anticoagulant and antithrombotic agent, this GalAG has been suggested as an emerging alternative for clinical use [72]. However, little was known about its hemorrhagic effect. It is important to highlight that heparin, a GAG of great clinical importance as an anticoagulant agent, presents risks of hemorrhage which varies from patient to patient [127,128,129,130]. In view of this fact, investigations into the hemorrhagic effect of DS were fundamental. Thus, Fernandez et al. compared the antithrombotic, hemorrhagic and anticoagulant effects of heparin and DS in rabbits. The results showed that DS is less hemorrhagic than heparin at equivalent antithrombotic doses [114]. Due to its reduced hemorrhagic potential, DS has been proposed as a safer alternative than heparin for use during hemodialysis in patients with renal impairment [131].

The main structural requirements of DS for its anti-IIa activity mediated by HCII, such as levels of sulfation, content of IdoA units and the minimal chain length for DS binding to HCII and formation of the DS:HCII:IIa ternary complex, were already uncovered by studies in the groups of Linhardt and Tollefsen [124,132,133,134]. Fractions of DS with higher content of IdoA are more active as antithrombotics [135], probably by enhanced flexibility of the IdoA monosaccharide residues. To elucidate the structural and conformational features involved in the formation of the active intermolecular complexes, mainly with HCII, underlying anticoagulant and antithrombotic mechanisms of action of DS [113], studies based on computational methods were recently performed to comprehend the advanced details behind the allosteric mechanisms of the DS-HCII interaction [17].

A screening of a virtual library gave rise to several topologies that were predicted to bind in the heparin-binding site of HCII at a ∼60° angle to helix D, a novel binding mode observed so far for the DS:HCII complex. This new binding geometry supports ternary DS-HCII-IIa complexation through a template mechanism [17]. This proposal stands in contrast to the previously accepted allosteric mechanism in which DS binds only to HCII, displacing the amino-terminal acidic domain of HCII which then interacts with the anion-binding exosite I of IIa and accelerates the formation of the HCII-IIa complex [136].

#### 3.2.2. Wound Healing

DS is predominantly expressed in the skin and is released at high concentrations during wound repair to act as a key player during healing events [137,138]. A study by Penc et al. showed that DS released after injury is a potent promoter of fibroblast growth factor-2 (FGF-2) function [138]. In subsequent research, authors have shown that DS as modulator of the functions of FGF-7 and FGF-10 has the capacity to accelerate wound healing by stimulating proliferation and migration of keratinocytes [139,140]. In fact, DS is able to interact with several GBPs that can be involved in events related to wound repair, including but not limited to IIa [113], activated protein C [141], collagen [142], fibronectin [143], α2β1-integrin [144], defensins [145], interferon-γ [146] and transforming growth factor beta (TGF-β) [147]. All these interactions suggest possible mechanisms by which DS plays its role in healing. However, in addition to these distinct interactions, DS also participates in indirect interactions with multiple other plasma proteins related to tissue repair and wound healing [137].

#### 3.2.3. Inflammation

Another potential medical application of DS relies on its anti-inflammatory effect. Belmiro et al. reported that subcutaneous administration of DS can inhibit colon inflammation in rats and promote reduction of macrophage recruitment, as well as T cell and macrophage activation [148]. In another study of the same group, the authors investigated the effect of DS extracted from the swine intestinal mucosa on inflammation and fibrosis in kidneys of mice after unilateral ureteral obstruction [149]. DS treatments were observed to reduce collagen, monocyte chemoattractive protein-1 (MCP-1) and TGF-β chemotactic protein content, as well as myofibroblasts and accumulation of macrophages in the kidney. The results indicated a protective effect of DS against the progression of renal inflammation and of fibrosis in kidneys [149].

The anti-inflammatory role of DS was also investigated by Gruber et al. [150]. In this study, they described the mucoprotective effect of systemic DS treatment in a preclinical model of oral mucositis (a potential complication of radiotherapy treatment of head and neck cancers). DS was in this model a potent agent capable of modulating radiation-induced oral mucositis. Further analysis was particularly made of protein expression associated with mucositis and the signaling changes involved in this complication. In all, epithelial morphology, proliferation and signaling were evaluated over two weeks of fractional irradiation with and without systemic administration of DS. The radioprotective effect of DS appears to be mainly based on an increase in epithelial junctions, thus strengthening epithelial integrity and reducing cellular loss. The authors pointed out that reduction of inflammation promoted by DS administration and local hypoxia may have contributed to mucoprotective activity [151].

#### 3.2.4. Cancer and Metastasis

Because DS is a key player in several evolutionary stages of cancer (tumorigenesis, growth and spread) [26,152], exogenous DS was studied regarding its potential property as anticancer agent, particularly to inhibit metastatic processes [153]. In the work of Kozlowski et al. [153], the ascidia DSs from *S. plicata* (Figure 5A) and *Phallusia nigra* (with similar structure to *A. nigra*, Figure 5B) were investigated regarding their ability to bind to selectins, blocking therefore the functions of these co-receptors during attachment of cancer cells in events of tumor spread and invasion. The assays were performed in wild type mice and with P-selectin deficient animals. What the study has shown was that DS molecules extracted from both tunicate species attenuated the metastasis in wild-type mice; however, in the tests performed with P-selectin deficient mice they did not show a significant effect. This suggests that the specificity of P-selectin inhibition is the primary mechanism by which DS plays in its anti-metastatic activity [153]. The key roles of GAGs in cancer process as well as potential targets for drug development have been extensively reviewed by Afratis et al. [152]. This reference is a useful source of information on the molecular basis of cancer-related properties of DS.

#### 3.2.5. Antiviral Activity

Besides the above-reported activities, the GalAG DS has also shown antiviral activities. In the work of Di Caro et al. [154], fractions of DS and CS were chemically oversulfated and tested regarding their properties against human immunodeficiency virus type 1 (HIV-1), herpes simplex virus type 1 (HSV-1) and human cytomegalovirus (HCMV). The assays performed revealed that while a 23.4 kDa oversulfated DS exhibits an EC_50_ (EC_50_ = concentration of drug that induces half of the maximum effect) of 0.04 μg/mLin HIV-1 inhibition, a second oversulfated DS of MW of 25 kDa showed activity against HSV-1 with EC_50_ of 0.01 μg/mL. The oversulfated CS of MW 17.3 kDa was the strongest anti-HCMV agent, showing its EC_50_ of 0.4 μg/mL [154].

## 4. FucCS

### 4.1. Overview

**Figure 6:**
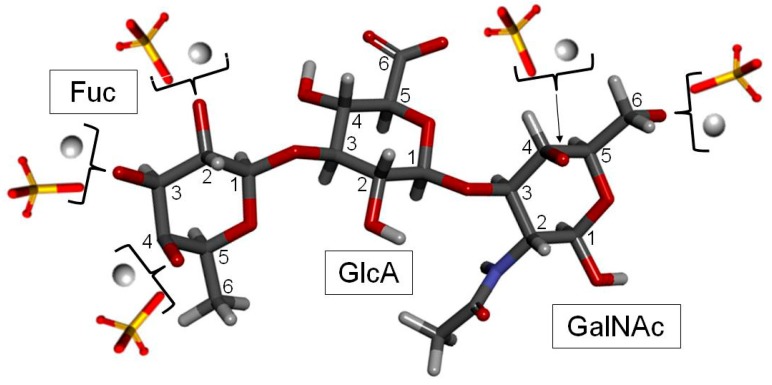
Representative trisaccharide unit {→4)-[α-L-Fucp-(1→3)]-β-d-GlcA-(1→3)-β-d-GalNAc-(1→} of the FucCS backbone from sea cucumber in its 3D stick models. The sites of possible substitution by sulfate groups are C2, C3 and C4 of Fuc*p* unit and C4 and C6 of GalNAc as indicated in the structure. Hydrogens, carbons, oxygens and nitrogens are represented in light grey, dark grey, red and blue, respectively. Carbon atoms are numbered according to their positions in the ring. Monosaccharides are indicated accordingly. Model created by the online AMBER/GLYCAM-based carbohydrate builder [40].

FucCS is a unique GalAG found in marine invertebrates such as sea cucumbers [6], crab [4] and octopus [61]. It plays a role as a structural component of the holothurian body wall and cartilage tissues of crab and octopus. In terms of structure, FucCS is composed of the same CS structure as vertebrates (Figure 1A, Figure 2 and Figure 6), although with additional branches of sulfated fucopyranose (Fuc*p*) units (Figure 1B). FucCS is then chemically described as {→4)-[α-L-Fucp-(1→3)]-β-d-GlcA-(1→3)-β-d-GalNAc-(1→}_n_ (Figure 6). The Fuc*p* unit may bear *O*-sulfation at positions C2 and C4, according to the holothurian species (Figure 1B and Table 3). Although sulfation patterns of the CS backbones may vary among different FucCSs, the E type sulfation (4,6-di-sulfation) seems to dominate [6,155,156]. Other unusual types of branches can also occur in CS. These rarer branches may include fructose in the case of the k-4 uropathogenic *Escherichia coli* [157], glucose in squid CS [158], a KS disaccharide [159], and the sulfated carbohydrate-protein linkage region in inter-α-trypsin inhibitor in human plasma, bikunin [160,161].

Structure determination of many holothurian FucCS via NMR spectroscopy has shown the presence of 4,6-di-sulfated, 4-mono-sulftated, 6-mono-sulfated and non-sulfated GalNAc units in the CS backbone [162]. In addition, a minor degree of 3-*O*-sulfation of GlcA can occur [163]. These proportions may vary with the species of holothurians [6,164]. Hence, structural heterogeneity of CS backbones can be observed among FucCS molecules extracted from the same species or among different species. The variation of sulfation patterns on the branching Fuc*p* units seems to be more relevant in terms of biological activity than the variation of sulfation on the main backbone. This is because the presence of the branching units on FucCS molecules provides this invertebrate molecule with the capacity to modulate numerous physiological systems. The levels of activity in these systems are not exclusively dependent on fucosylation but also on sulfation patterns of the branching units (Table 3). Below, we discuss in detail the potential biomedical functions exerted by FucCS in multiple pathophysiological systems. The structural motifs responsible for the best activity are also discussed when known. These biologically active structural motifs are very frequently the sulfation patterns of the branching Fuc*p* unit.

### 4.2. Biomedical Properties

#### 4.2.1. Coagulation and Thrombosis

CS is not active as an anticoagulant, but fucosylation renders it active depending on the pattern of sulfation on the fucosyl branch (Table 3) [164]. Among the multiple potential biomedical applications of FucCSs, their potential anticoagulant properties are the most desirable and best studied. Previously, the anticoagulant/antithrombotic mechanism of action of FucCS had been believed to be centered primarily on being a catalyst for the inhibitory functions of the serpins antithrombin (AT) and HCII, and for the coagulation serine-proteases, IIa and Xa [19]. The ability of FucCS to potentiate IIa inhibition is mainly characterized by the activity of HCII [185], and the sulfation degrees have been shown to play a crucial role in this serpin-dependent activity [186]. More recently, however, a serpin-independent anticoagulant/antithrombotic activity has been assigned to holothurian FucCS [187,188,189,190,191], and the mechanism of action behind this unique property has been reported to rely on the capacity of the FucCS to impair factor Xa generation by inhibiting the formation of the intrinsic tenase complex, which is in turn made by factors VIIIa, IXa and X [156,187,191,192]. This serpin-independent anticoagulant activity of FucCS has been reported to dominate the process, and the serpin-dependent activity seems to be just residual in the systemic point-of-view [191].

Since non-fucosylated CS is inactive in anticoagulation, the removal of the Fuc*p* branching unit from the FucCS is expectedly detrimental to anticoagulant and antithrombotic activities [19,21]. In addition to the presence of the sulfated fucosyl branch, the sulfation pattern of this branch was also reported to be a contributing factor [6,164,186]. A Fuc*p* unit with the 2,4-disulfation pattern was reported to be the anticoagulant/antithrombotic motif [193]. The concentration of 2,4-*O*-disulfated Fuc*p* units in the FucCSs of the four holothurian species *Isostichopus badionotus*, *Thelenota ananas*, *Stichopus japonicus*, and *Apostichopus japonicus* are higher when compared to the molecules of the other species (Table 3). Although this concept of the 2,4-di-sulfation is highly supported based on several studies [165,166,186,193], a new proposition defends the conception that different structures (different sulfation patterns) of the branching α-L-Fuc*p* units do not have an impact on the anticoagulant outcome [173]. Procoagulant effects from low doses of the holothurian FucCS, as measured by thrombin generation, have also been reported [194], adding to the range of contradictions. Dual and antagonic biomedical effects of sulfated glycans, including those of marine source, are not unexpected [195].

#### 4.2.2. Hemodialysis

Minamiguchi et al. explored the anticoagulant property of the FucCS from *S. japonicus* (MW around 14 kDa) in hemodialysis [34,196]. As opposed to unfractionated heparin (UFH) and low molecular weight heparin (LMWH), which showed significant bleeding time, nafamostatmesilate (FUT) and the FucCS have just scarcely prolonged bleeding time. As opposed to FUT, which was able to increase only the activated partial thromboplastin time (aPTT), the FucCS was able to increase both aPTT and thrombin clotting time. This indicates that the studied FucCS can be used as an anticoagulant agent in procedures of hemodialysis accompanied by low risks of hemorrhage [34]. FucCS has also shown an AT-independent anticoagulant mechanism of action in the hemodialysis experiment [196]. This mechanism helps to explain the low hemorrhagic risks of the low MW FucCS tested in the in vivo experimental models of hemodialysis as compared to the other standards, including UFH and LMWH [196].

#### 4.2.3. Atherosclerosis

FucCS from sea cucumber species *Ludwigothurea grisea* has shown great affinity for lipoproteins, especially apo B-containing lipoproteins like low-density lipoprotein (LDL) and very low-density lipoprotein [197]. The binding affinity for LDL increases proportionally to MW of *L. grisea* FucCS and is lost upon desulfation and defucosylation. Since LDL is largely responsible for the development of atherosclerosis or any other disease related with the obstructions of the coronaries, and because FucCS has great affinity to LDL, application of FucCS seems promising in capture and decrease of LDL plasma content [197]. The reasonable lifetime of FucCS in the plasma when orally administered supports the medical use of holothurian FucCS through the oral route [198]. In another study, FucCS from *S. japonicus* has also indicated the potential medical benefits of this invertebrate GalAG in atherosclerosis [199]. The authors postulated that FucCS has the capacity to inhibit neointimal thickening induced by balloon catheterization. The mechanism of action proposed for FucCS in the event of atherosclerosis relies on its capacity to inactivate growth of aberrant smooth muscle cells (SMC) in the system.

Sulfation patterns of fucose branches were proved to affect the anti-hyperlipidemic activity of FucCS [200]. The anti-hyperlipidemic effects were compared using a high-fat high-fructose diet-fed C57BL/6J mice model. Both FucCS from *I. badionotus* and *Pearsonothuria graeffei* showed significant effects on lipid profile improvement, liver protection, blood glucose diminution and hepatic glycogen synthesis. Specifically, FucCS from *P. graffei* with 3,4-*O*-disulfation fucose branches (Table 3) was more effective in reduction of blood cholesterol, LDL and atherogenic index. These results clearly indicate that FucCS from *P. graffei* could be used as a potential anti-hyperlipidemic agent.

#### 4.2.4. Cellular Growth

The effects of FucCS from *L. grisea* on vascular SMC from rat thoracic aorta and on human umbilical vein endothelial cell (HUVEC) in culture, in the presence or absence of FGF-1 and FGF-2, were examined [35]. While FucCS showed an efficient inhibitory activity on SMC proliferation, no effects have been observed on HUVEC proliferation and migration assays without FGF exogenously added to the culture media. The presence of sulfated fucose branches on FucCS was indicated to be an essential determinant to the activity of the holothurian GalAG on cell proliferation and migration; upon defucosylation, the effects of FucCS on cells are abolished [35].

#### 4.2.5. Angiogenesis

The background concerning the effects of FucCS on cellular growth was re-examined by Tapon-Bretaudière et al. with respect to the effects of the holothurian GalAG in angiogenesis [32]. The effects of the FucCS from *L. grisea* were investigated on endothelial cells through an in vitro angiogenesis-related experimental model utilizing Matrigel. In the presence of FGF-2, FucCS showed the capacity to induce formation of vascular tubes by endothelial cells in Matrigel. The vascular tubes were observed distributed as very organized capillary-like networks within typical closed structures. Heparin did not promote vascular tube formation in this experimental model of angiogenesis indicating therefore that the structural features of the sulfated glycans play a role in the outcome. As in most of the other biomedically relevant actions, desulfation and defucosylation abolish the pro-angiogenic effects of FucCS [32].

#### 4.2.6. Fibrosis

Melo-Filho et al. have studied the properties of FucCS from *L. grisea* in respect to its capacity of retarding or preventing the pathological formation of renal fibrosis. The method utilized for this was an in vivo model of rats submitted to unilateral ureteral obstruction (UUO) [36]. Compared with control animals (FucCS-untreated rats), collagen deposition decreased in animals treated with FucCS. The cell amounts of myofibroblasts and macrophages were also reduced. Another beneficial factor of FucCS was its capacity to diminish the production of transforming growth factor (TGF)-β. As observed in P-selectin-deficient mice submitted to UUO, renal fibrosis was observed to be greatly attenuated. Interestingly, these deficient mice showed also little response to the *L. grisea* FucCS. As conclusion, FucCS is capable of attenuating renal fibrosis on UUO-submitted animals, and this effect seems to occur through a P-selectin-related mechanism of action [36].

#### 4.2.7. Cancer and Inflammation

Like heparin, which has the capacity to inhibit P- and L-selectin binding to their ligand, sialylLewis^X^, resulting consequently in antitumor and anti-inflammatory outcomes, the FucCS from *L. grisea* was analyzed regarding its antiselectin property, also in the context of presenting potential antitumor and anti-inflammatory activity [24]. Indeed, like heparin, FucCS was observed to exert a potent inhibition effect of both P- and L-selectin bindings to immobilized sialylLewis^X^ and attachment of carcinoma cell (LS180) to immobilized P- and L-selectins. The FucCS inhibition process occurs in a dose-dependent manner. FucCS showed also the capacity of inhibiting adenocarcinoma MC-38 cells to colonize lungs in an experimental model of metastasis in mice [24]. In addition to this activity, the authors also demonstrated through two different models of inflammation (thioglycollate-induced peritonitis and lipopolysaccharide-induced lung inflammation) that *L. grisea* FucCS showed the capacity to inhibit neutrophil recruitment. Both inhibitory properties are dose-dependent, and the effective doses gave no alterations in the plasma aPTT. This indicates that FucCS showed its antitumoral or anti-inflammatory response without presenting hemorrhagic risks [24]. Panagos et al. also investigated this antiselectin capacity of FucCS from *Holothuria forskali* by screening FucCS-derived oligosaccharides via microarray techniques [182]. In this study, the authors have proposed the structural conformation of the FucCS building blocks and the relevance of sulfation patterns of the Fuc*p* units in modulating the overall 3D structure of this GalAG in solution.

Zhao et al. observed by both in vivo and in vitro assays that FucCS can concomitantly reduce levels of both metastasis using B16F10 cells of mouse melanoma, and tendency to thromboembolism when FucCS is administered. It has been shown that under the holothurian GalAG administration, down-regulation of both transcription and protein expression of tissue factor can be achieved, and fibrin formation is inhibited primarily by attenuating the generation of activated factor Xa. In addition to these activities, FucCS also has the capacity to suppress activation of p38MAPK and ERK1/2 signaling pathways. These signaling pathways are considered the central routes for the expression of metastasis-related matrix metalloproteinases [201]. These findings suggest that a single GalAG molecule can exert dual medical properties, as both anticancer and antithrombotic agent. In a separate publication by He et al., the investigators found that the FucCS from *I. badionotus* could markedly inhibited tumor growth by down-regulating expressions of Hif-1α (hypoxia-inducible factor 1-alpha), heparanase, and vascular endothelial growth factor in vivo [202].

#### 4.2.8. Microbial Infections

##### Viral Infections

The anti-HIV activity of the FucCS from *T. ananas* has been studied [29,30]. Data suggested that this FucCS can effectively and selectively block both X4- and R5 × 4-tropic HIV-1 infection of C8166 cells, as well as peripheral blood mononuclear cells with little cytotoxicity against these cells. Results indicated that this FucCS can bind to the HIV membrane glycoprotein 120 (gp120) at nanomolar binding affinity via molecular interaction with the CD4i epitope. As expected, based on all actions of FucCS examined so far, the sulfated fucosyl branches were indicated to be structural motifs responsible for anti-HIV-1 activity. In addition, molecular size was also observed to participate given the fact that several FucCS-derived low MW fragments have exerted higher anti-HIV-1 and lower anticoagulant activities than heparin and the native FucCS used as standards [29]. The primary mechanism of action underlying the anti-HIV outcome of FucCS was reported to be on blocking the virus entry into the host cells. This mechanism of action is in good accordance with the general antiviral mechanism of action proposed for sulfated polysaccharides [203].

##### Malaria-Related Protozoan Infection

FucCS also modulates infections caused by the protozoan parasite *Plasmodium falciparum* [33]. Sequestration of *P. falciparum*-infected erythrocytes (Pf-iEs) in the microvasculature of vital organs is an essential event during *P. falciparum*-caused pathogenesis, such as cerebral malaria and malaria in pregnancy. These events are driven by cytoadhesion of Pf-iEs onto GAG receptors displayed at the surface of host endothelial cells, of non-infected erythrocytes, and on placenta trophoblasts. Based on the general background concerning the antimicrobial activity of sulfated glycans [203], exogenous GAGs can be used to interfere with the host GAG-Pf-iEs interactions as a therapeutic strategy against the malaria complications. On this basis, the use of FucCS isolated from *L. grisea* has shown the ability to inhibit Pf-iEscytoadhesion [33]. The holothurian GalAG is also capable of blocking parasite development by interfering with the merozoite invasion of the *P. falciparum*. The inhibition of protozoan infection is abrogated when the Fuc*p* branches are removed [33].

#### 4.2.9. Hyperglycemia Diabetes-Related Processes

##### Diabetic Nephropathy

GAG-based drugs such as LMWH can be renoprotective in diabetic nephropathy. In this regard, FucCS from *L. grisea* has been tested for its clinical potential to prevent or diminish the pathological consequences related to diabetic nephropathy [38]. Diabetes mellitus was experimentally induced by streptozotocin in male Wistar rats to perform the tests. FucCS and LMWH were both administered subcutaneously. Blood glucose, blood pressure, albuminuria, and renal function were monitored after 12-day treatment. Kidneys were evaluated for mesangial expansion and collagen content. Immunohistochemical quantifications of macrophages, TGF-β, nestin and immunofluorescence analysis of heparanase-1 and glomerular basement membrane, heparan sulfate content were also examined. Gene expression of proteoglycan core proteins and enzymes involved in GAG assembly/degradation were analyzed by TaqMan real-time PCR [38]. Treatment with GAGs prevented albuminuria but did not change the glucose levels or other functional aspects. The negative group exhibited increased mesangial matrix deposition and tubulointerstitial expansion, but these aspects were prevented by administration of both GAG types. TGF-β expression and macrophage infiltration were also prevented by both GAGs, and podocyte damage was halted. The diabetic milieu resulted in the downregulation of agrin, perlecan and collagen XVIII mRNAs, along with the expression of enzymes involved in GAG biosynthesis. It was observed that FucCS and LMWH treatments could positively modulate such changes. Heparanase-1 expression was significantly reduced after GAG treatment without affecting the globular basement membrane and heparan sulfate content. These findings suggest that FucCS could act as a protective agent against the aggravating consequences of nephropathy during diabetes at similar levels of response seen for LMWH [38].

##### Diabetes-Related Apoptosis of Islets of Langerhans

Apoptosis of pancreatic islets of Langerhans is a common characteristic of diabetes. High-fat high-sucrose diet-induced insulin-resistant mice were studied for 19 weeks with and without FucCS treatment [204]. It was shown that FucCS treatment significantly decreased fasting blood glucose, insulin, and TNF-α levels. FucCS treatment also increased the serum levels of adiponectin, improved insulin resistance, and repaired HFSD-injured islets of Langerhans. FucCS supplementation showed the ability to inhibit pancreas cell apoptosis via reduction of cytochrome c, caspase 9, and caspase 3 mRNA expressions, cytochrome c release into the cytoplasm, and caspase 9 and cleaved caspase 3 protein expressions. These effects were associated with up-regulation of Bcl-2 and Bcl-xL mRNA and protein expressions, and down-regulation of Bax and t-Bid mRNA and protein expressions. Treatments achieved by FucCS combined with rosiglitazone showed a synergistic protection of pancreatic islets. Overall, these data show that improvement in insulin resistance and protection of islets of Langerhans from apoptosis can be achieved by FucCS via inactivation of the intrinsic mitochondrial pathway [204].

#### 4.2.10. Tissue Damage

Monteiro-Machado et al. showed that FucCS from *L. grisea* can present potential therapeutic properties against myotoxicity induced by snake venom from *Bothrops jararacussu* [205]. Myotoxicity was induced in muscle tissue in vitro by crude venom, by bothropstoxin-I alone or by bothropstoxin-I and bothropstoxin-II combined. The myotoxicity was abrogated in all cases in the presence of FucCS. It has also shown that FucCS can inhibit edematogenic activity and partially prevent the reduction of total leukocytes in blood when pre-incubated with crude venom. Taking together all data presented by reference [205], it has been shown that FucCS is a reliable inhibitor of myotoxicity and inflammation induced by *B. jararacussu* venom or the related toxins.

## 5. Drawbacks

### 5.1. The Dilemma Regarding Effectiveness and Safety of CS as a Dietary Supplement for Treatments of Osteoarthritis

CS has been recommended for use in symptomatic relief and possible treatment for osteoarthritis, often in combination with glucosamine, since the mid 1900s [206]. It has been hypothesized that increasing concentrations of CS will allow for regeneration of the cartilaginous tissues. However, a defined mechanism of action has not been described [206]. Because of this lack of clear efficacy, CS has been under scrutiny for whether it is in fact beneficial in treatment of osteoarthritis. Guidelines were set by the American College of Rheumatology for knee osteoarthritis [207] and by the Osteoarthritis Research Society International [208]. Neither entity explicitly condones the use of CS. Much of the experimental data collected in vitro that identifies bioactivity of CS relevant to efficacy in treatment of osteoarthritis are based on CS concentrations far higher than are achievable in the circulation [206]. Understanding the structural requirements for bioactivity might lead eventually to structurally better-defined and less heterogenous CS-based medicinal natural products.

In addition to some of these issues, there are also some considerations to make concerning patients who are taking CS for relief of osteoarthritis, especially in comparison to those who are administered more well-defined medicines like celecoxib. In comparison with placebo, for example, patients with osteoarthritis taking CS reported a reduction in pain [207,208]. However, other surveys of patients have found the opposite, showing no advantage over placebo [209]. This indicates that CS may be either effective or ineffective in the relief of pain and osteoarthritis. This variation in effectiveness may be a result of patient to patient variation, and/or to the different levels of pain and osteoarthritis. What is certainly clear is that more in-depth studies must be conducted to determine the safety and extent to which the effects are clinically achieved and observed and, at the end, statistically registered.

Another point to be clarified is the efficiency of glucosamine in combined administration with CS. Although evidence for the symptomatic efficacy of glucosamine added to CS preparations for treatments of knee osteoarthritis have been seen, for example, in the study of Zeng et al. [207], pain relief following ingestion of CS has been reported to be independent of simultaneous or prior intake of glucosamine, as stated in the work of Jackson et al. [210]. In this study, the authors stated that although combined dosing of glucosamine with CS was found to reduce the plasma levels of glucosamine, any improved pain relief by combined administration of the two components cannot be explained by higher circulating concentrations of glucosamine.

### 5.2. The Presence of Unexpected Components in CS-Based Pharmaceutical Formulations for Oral Administration

Of great concern with the application of CS are potential issues associated with the presence of non-desirable structures or components during the manufacturing processes of CS as this GalAG is extracted from a wide range of sources [211]. Because CS is expressed and isolated as long chains, with high structural diversity with respect to sulfation patterns containing just a single chain, it is important to highlight the off-target effects that may be associated with the structural heterogeneity. Further unexpected effects may arise from the presence of another GAG type present in the preparations, such as KS [212]. KS is a structurally complex GAG that can potentially give rise to unexpected effects when administered accidentally during common usage of CS [213] although any deleterious effects have not thus far been demonstrated [65].

In an industrial setting, it is not uncommon for these impurities to be found in the final product due to similarities within both isolation and purification procedures of CS and KS [211]. KS contamination can therefore be expected in CS formulations for multiple usages, ranging from laboratory reagents to its use as an alternative medicine [212]. In some cases, this contamination of KS may be remarkably high, with the total amount being up to 16% in the final product [65]. This may result both in possible negative attributes within the formulation and label claims, as well as possible dishonest representation of the product on the market [65]. This contamination, however, is not limited to KS, as other GAG types, protein and nucleic acids may also be found in some of the CS formulations [211]. In addition to the presence of non-desirable components, there is also the possibility of occurrence of disulfated residues in final formulations [214]. This is particularly likely to occur when derivatives from marine sources are used. This whole issue clearly highlights the importance of source material versus the final product composition that reaches the consumer [214]. This issue becomes especially important in the cases of nutraceutical preparations of CS that are not subject to the same quality standards as pharmaceutical formulations of drugs, which must follow rigorous processes of quality assurance and control. In some cases, the stated purity of CS is not even found or offered [54,214].

Nutraceutical formulations of CS could also be subject to intentional adulteration to reduce the initial cost involved in the production of the active ingredient. This practice is thought to have played a major role in the 2008 “Heparin crisis” and resulted in potent adverse effects to the end consumers [211]. The discrepancy between pharmaceutical and nutraceutical preparations can also go as far as a noticeable difference in expected therapeutic effects. The efficacy of pharmaceutical-grade CS preparations should not be extrapolated to nutraceutical formulations [215] for which controls are usually less stringent, depending on the local jurisdiction.

### 5.3. Potential Antigenic and Autoimmune Side-Effects of CS

The structural heterogeneity of GAGs and likely GalAGs gives a diversity of sequences for interaction with GBPs, and consequent regulation of multiple GBPs. This consequently enlarges the spectrum of biological actions of these sulfated glycans and makes them important structural players in perhaps all physiological systems of the body. Conversely, this same structural heterogeneity can give rise to issues related to possible antigenic effects. Binding epitopes that have been associated with serious autoimmune disorders may be involved in interaction with GAGs (and likely GalAGs). One of interest is the human natural killer-1 (HNK-1) epitope, composed of the 3-sulfated GlcA [216], which is characteristic of CS-K, CS-L and CS-M (Table 1). The HNK-1 epitope is found within the myelin sheaths and is linked to autoimmune disease in the peripheral nervous system. It is recognized by its conjugate monoclonal antibody anti-HNK-1 [217]. Interesting, the rare 3-sulfated GlcA unit in intact CS chains is also recognized by anti-HNK-1 [218]. Hence, this raises the question of potential triggering of autoimmune complications by CS-K, CS-L, CS-M and some FucCS.

Regarding this same issue regarding the possible antigenicity of CS, it is well-known that platelet factor 4 (PF4)/heparin complexes can trigger the immunological response that will cause heparin-induced thrombocytopenia (HIT) [219,220]. In this event, the intermolecular complexes aggregate and will trigger the production of antibodies associated with the disease state. Furthermore, PF4 has shown binding properties to CS [221,222] and its oversulfated version [223], therefore leading to high-level production of HIT-related antibodies in patients undergoing CS-based treatment [224]. For these reasons, these GalAGs may also be capable of inducing thrombocytopenia in a similar manner as heparin does in HIT. This side-effect will, of course, be dependent on the concentration of the epitope. Thus, sulfation patterns of CS, chain heterogeneity and concentration are all factors that play a role in the antigenic events of CSs.

### 5.4. Highly Sulfated CS can Activate the Kinin-Kallikrein System

As in the “*Heparin crisis*” of 2008 [64,225,226,227,228,229], highly sulfated CS from either from natural sources like CS-D, CS-D, CS-K, CS-L, and CS-M (Table 1) or from chemical reactions [230] are likely to be capable of triggering the same side effects as oversulfated chondroitin sulfate (OSCS) in the activation of factor XII and kinin-kallikrein system, which will result in bradykinin formation and severe hypotension. The ability of highly sulfated CS to activate the kinin-kallikrein system is likely to depend on both the extent and the pattern of sulfation [230].

### 5.5. Potential Problems for the Medical Use of DS and other GalAGs

The potential downsides of the medical use of DS are like those of CS; it is appropriate to discuss here some aspects common to all the GalAGs. The use of tissues from cows and pigs as main sources of raw materials for DS and other GalAGs can, in principle, lead to the potential contamination of potentially harmful and undesirable molecules if the original source is not processed well. This is not a current risk, but problems could arise mainly from suboptimal techniques in the purification processes of GalAGs as a whole, as these glycans are usually extracted as byproducts from the large-scale agricultural exploration of farmed animals. There are three ways to specifically overcome this issue. First would be to ensure that the manufacture of medical grade GalAGs meets the same exacting standards as current production methods for heparin [231]. The second alternative could be the use of other sources of DS, which may not contain, or contain in much lesser amounts, the contaminants of the mammalian tissues. These new sources are the marine organisms [107]. The third and seemingly last alternative would be the use of chemically synthesized DS from the laboratory [107,232].

The second concern in medically exploring DS might be the undesirable side-effects of this molecule when exogenously administered. It is known that DS plays a role in multiple physiological systems, and the administration of DS in pharmacologically active doses for a certain type of health complication could potentially also lead undesirably to detrimental impairments of other key functions of DS in other systems. Comparisons with the widespread use of the multivalent GAG heparin as an antithrombotic agent are reassuring but should not lead to complacency. A rational way to overcome this problem would be the recognition of specific sulfation patterns that are selectively important for only one or a few complications and the use of exogenous DS with a particular sulfation pattern, so that a selective drug could be developed, and the specific type of treatment could be reached.

The third concern about the medical use of exogenous DS would be the potentially dangerous harmful effects of certain DS derivatives, especially the oversulfated versions tested for antiviral activity [154]. As documented above, oversulfated DS is likely to trigger the same side effects of highly sulfated CSs and OSCS regarding the activation of factor XII and kini-kallikrein system-dependent hypotension. As opposed to CS in which experiments were performed to prove the side-effects of highly sulfated CS [230], no tests have been performed to prove such a hypothesis for highly sulfated DS. If it is confirmed that these undesirable actions can be induced by oversulfated DS, these GalAG derivatives are very unlikely to be employed as future drugs for any of their biomedical functions because they will not fulfill the minimal requirements for safety.

The fourth concern regarding the medical application of some DS structures is related to the autoimmune response related to PF4, which may lead to thrombocytopenia or HIT [233]. Because naturally or artificially oversulfated DS structure resembles the one of OSCS, DS can also trigger HIT, although the regular DS (CS-B) structure, as used as active ingredient of danaparoid, is used in treatments of patients prone to HIT [234,235]. As for CS, this side effect might be dependent on concentration and structural heterogeneity. On the other hand, DS will probably not be as antigenic as CS in the case of HNK-1 because, as opposed to the latter, which can contain the antigen 3-sulfated GlcA epitope, DS does not present this motif (Table 2). Nonetheless, while not confirming the hypotensive side-effect, there are studies that indicate that oversulfated DS can cause kallikrein activation [236,237].

### 5.6. Potential Harmful Effects of FucCS

FucCS is the GalAG that exerts the highest number of biomedical functions. These activities include anticoagulation and antithrombosis; beneficial effects in hemodialysis, atherosclerosis, cellular growth, and angiogenesis; in fights against fibrosis, tumor growth, inflammation, viral and protozoan infections; beneficial effects in hyperglycemia, in diabetes-related pathological events and in snake venom-caused tissue damage. FucCS would comprise a potent and promising medical agent to be explored in the future. However, obstacles such as potentially harmful side-effects and unclear routes of synthesis make its real implementation as a drug candidate difficult, therefore hindering its use in studies of clinical trials and the future application of this marine GalAG in medicine.

Although FucCS shows good effectiveness as opposed to CS’s use in osteoarthritis, and relative safety since it is extracted from a non-harmful sea organism, the greatest obstacle of all would be the similar harmful effects reported for OSCS. In this case, the harmful effect resides in the capacity of highly sulfated glycans such as FucCS to activate coagulation blood factor XII [238,239] and consequently induce kinin-related severe hypotension caused by kallikrein, a potent regulator of blood pressure via activation of bradykinin, besides inducing platelet aggregation [186] and spontaneous bleeding in humans [188]. Unfortunately, the real use of FucCS in the pharmaceutical industries is still some years away. Nevertheless, an enormous amount of recent research concerns the potential medicinal properties of this molecule [6,164,240].

## 6. Concluding Remarks

Medical interest in GalAGs has expanded worldwide, especially with the boom in projects related to medical glycomics. The principal types of GalAGs are CS, DS and FucCS. Sub-types also exist and are primarily characterized by different patterns of sulfation. Screenings have shown positive effects of GalAGs in multiple biomedical areas, including but not limited to thrombosis, cancer, inflammation, neurological disorders and microbial infections. However, the applications of GalAGs in medicine have been questioned. Doubts center primarily on their real effectiveness and potential harmful effects. There are contradictory opinions regarding the benefits of CS-based pharmaceutical formulations used for arthritis as well as the safety conditions in manufacturing processes of GalAGs, as GAGs, during the steps of isolation and purification for the global market. Impurities such as the KS content have also been reported in CS-based pharmaceutical preparations. More important are the harmful side effects of FucCS, including OSCS in activation of factor XII and kinin-kallikrein system-dependent hypotension. Despite the great number of potential biomedical applications and the diversity of chemical structures, the downsides of GalAGs considerably restrict the other medical exploitations of GalAGs, in addition to the use of CS as dietary supplements destined for aiding complications related to joint pain/inflammation and osteoarthritis. As clear synthetic routes of biomedically active GalAGs’ oligosaccharide sequences are not yet fully practical, and the mechanisms of action concerning their intermolecular complexes with GBPs are not completely understood, the current and future medical applications of GalAGs will continue to be limited. Protocols employed during large-scale manufacture must be also optimized. We believe all these areas related to the science and medical exploration of GalAGs can significantly develop in the next few years, especially accompanied by the incentive and parallel progress of medical glycomics worldwide.

## Figures and Tables

**Table 1:** Common and novel CS disaccharide units described by sulfation patterns and by common marine sources.

Type	Structure	Source	Reference
CS-A	GlcA(β1−3)GalNAc(4OSO_3_^−^)	Whale	[58]
CS-C	GlcA(β1−3)GalNAc(6OSO_3_^−^)	Shark	[59]
CS-D	GlcA(2OSO_3_^−^)(β1−3)GalNAc(6OSO_3_^−^)	Fish, Shark, Squid	[47,52]
CS-E	GlcA(β1−3)GalNAc(4,6diOSO_3_^−^)	Squid	[46,60]
CS-K	GlcA(3OSO_3_^−^)(β1−3)GalNAc(4OSO_3_^−^)	King Crab and Octopus	[46,61,62]
CS-L	GlcA(3OSO_3_^−^)(β1−3)GalNAc(6OSO_3_^−^)	Squid	[46]
CS-M	GlcA(3OSO_3_^−^)(β1−3)GalNAc(4,6diOSO_3_^−^)	Squid	[46]

**Table 2:** Common and novel DS (CS-B) disaccharide units described by sulfation patterns and by common sources.

Type	Structure	Source	Reference
DS (4S)	IdoA(α1−3)GalNAc(4OSO_3_^−^)	Bovine and porcine mucosa	[108]
DS (2S4S)	IdoA(2OSO_3_^−^)(α1−3)GalNAc(4OSO_3_^−^)	Tunic of ascidian	[104,109]
DS (6S)	IdoA(α1−3)GalNAc(6OSO_3_^−^)	Bovine liver, bovine spleen, rabbit liver and hog spleen	[58]
DS (2S6S)	IdoA(2OSO_3_^−^)(α1−3)GalNAc(6OSO_3_^−^)	Tunic of ascidian	[105]
DS (2S4,6S) (CS-H)	IdoA(2OSO_3_^−^)(α1−3)GalNAc(4,6diOSO_3_^−^)	Hagfish skin and Hagfish notochord	[72,110,111,112]

**Table 3:** Sulfation patterns (proportions of the branching sulfated fucose units) of FucCS from 27 sea cucumber species analyzed so far.

Species	Fuc0S	Fuc3S	Fuc4S	Fuc2S4S	Fuc3S4S	References
*Ludwigothurea grisea* ^a^	0	−	~49	~20	~17	[19,165]
*Pearsonothuria graeffei*	−	−	81.6	18.4	−	[166]
*Holothuriava gabunda*	25.6	−	50.2	15.8	8.4	[166]
*Stichopus tremulus*	−	−	24.8	22.4	52.8	[166]
*Isostichopus badionotus*	−	−	4.1	95.9	−	[166]
*Thelenota ananas*	0	~25	~22	~53	0	[167,168]
*Stichopus japonicus* ^b^	18	17 ^c^	0	16	23	[169]
*Holothuria edulis* ^d^	−	−	Nd	18	Nd	[170]
*Apostichopus japonicus* ^d^	−	−	Nd	45	Nd	[170]
*Holothuria nobilis* ^e^	−	Nd	Nd	−	Nd	[170]
*Acaudina molpadioidea* ^f^	−	−	−	−	−	[171]
*Athyonidium chilensis* ^f^	−	−	−	−	−	[172]
*Patalus mollis*	0	0	26	34	40	[173]
*Massinium magnum* ^g^	_	_	_	_	Nd	[174]
*Apostichopus mauritania*	_	_	_	16.7	66.7	[175]
*Holothuria mexicana*	6.24	5.58	37.16	_	51	[176]
*Cucumaria djakonovi*	_	_	~25	~50	~25	[177]
*Eupentaca fraudatrix* ^h^	33.3	_	_	_	66.7	[178]
*Stichopus horrens* ^i^	_	_	_	Nd	_	[179]
*Stichopus chlorontus* ^i^	_	_	_	Nd	_	[179]
*Holothuria scabra* ^j^	_	_	27.1	72.9	_	[180]
*Holothuria tubulosa* ^k^	_	_	14.3	42.85	42.85	[181]
*Holothuria stellati* ^k^	_	_	20	40	40	[181]
*Holothuria forskai*	_	_	15	39	46	[182]
*Cucumaria frondosa* ^l^	_	_	_	25	62.5	[183]
*Cucumaria japonica*	_	_	_	20	80	[184]
*Actinopyga mauritiana*	_	_	_	20	80	[175]

^a^ The CS backbone of FucCS from *L. grisea* has been extensively characterized. It is composed of GalNAc units with the following substitution percentages: 12% 4,6-di-sulfated, 53% 6-mono-sulfated, 4% 4-mono-sulfated, and 31% non-sulfated [165]. Some 3-*O*-sulfation of GlcA has also been identified [163]. ^b^ The CS backbone of this FucCS was mostly characterized as CS-E [155], which is predominantly composed of 4,6-*O*-di-sulfated GalNAc units. ^c^ Not determined. ^d^ Although the mono-4S and di-3S4S fucosyl units have been assigned in the FucCS of *H. edulis* and *A. japonicus* in [170], the amounts of these units were not provided therein. ^e^ The FucCS from *H. nobilis* was studied by NMR but the anomeric signals belonging to the fucose residues were rather too weak and broad to allow integration and further quantitation of their proportions. However, mono-3*S*, mono-4*S*, and di-3*S*4*S*fucosyl units were clearly observed [170]. ^f^ Structures studied by Fourier transform–infrared spectroscopy. Just a few structural features were raised. The sulfation patterns of FucCS from these two holothurian species are still an unknown. ^g^ The CS backbone was characterized and found to be mainly composed of CS-E and CS-A at a ratio of 9:1, respectively [174]. ^h^ The CS backbone of FucCS from *E. fraudatrix* was characterized to be composed of a 1:1 ratio of CS-E to CS-A [178]. ^i^ Both FucCS from *S. horrens* and *S. chlorontus* were characterized with a backbone mostly composed of CS-E [179]. ^j^ FucCS from *H. scabra* was characterized with a backbone comprised mostly of CS-C with some CS-E and CS-A units [180]. ^k^ The CS backbone of FucCS from *H. tubulosa* was found to be composed of units of CS-E and CS-A at a ratio of 4:1 while the FucCS backbone from *H. stellati* is also composed of CS-E and CS-A in a ratio of 4.5:1 [181]. ^l^ The FucCS characterized from *C. frondosa* was found to have an unusual sulfated alpha-L-Fuc*p* linked to the O-6 of the GalNac residue, resulting in 12.2% of the sulfation pattern in addition to the sulfated fucose residues indicated in the table above [183]. Table modified with permission from [6].
